# Strategies for effective goals of care discussions and decision-making: perspectives from a multi-centre survey of Canadian hospital-based healthcare providers

**DOI:** 10.1186/s12904-015-0035-x

**Published:** 2015-08-19

**Authors:** Amanda L. Roze des Ordons, Nishan Sharma, Daren K. Heyland, John J. You

**Affiliations:** Department of Critical Care Medicine and Division of Palliative Medicine, University of Calgary, South Health Campus, 4448 Front St SE, Calgary, AB T3M 1M4 Canada; W21C (Ward of the 21st Century), Cumming School of Medicine, University of Calgary, GD01 TRW Building, 3280 Hospital Drive NW, Calgary, AB T2N 4Z6 Canada; Critical Care Programme, Queen’s University, 76 Stuart Street, Kingston, ON K7L 2V7 Canada; Departments of Medicine and Clinical Epidemiology & Biostatistics, McMaster University, 1280 Main Street West, Room HSC-2C8, Hamilton, ON L8S 4K1 Canada

## Abstract

**Background:**

Communication gaps impact the quality of patient care. Previous research has focused on communication barriers rather than seeking solutions. Our aim was to identify strategies for effective communication and decision-making about goals of care for medical interventions in serious illness, from the perspectives of hospital-based healthcare providers.

**Methods:**

A cross-sectional survey composed of closed- and open-ended questions about goals of care communication and decision-making was administered to healthcare providers in 13 centres in six Canadian provinces. We analyzed a portion of the open-ended survey questions, specifically (1) suggestions for overcoming barriers encountered in discussing goals of care, and (2) currently effective practices. Thematic content analysis was used to analyze responses to the open-ended questions.

**Results:**

Of the 1,256 respondents to the larger survey, 468 responded to the open-ended questions (37 %), including 272 of 512 nurses (53 %), 153 of 484 internal medicine trainees (32 %), and 43 of 260 attending physicians (17 %). Responses to each of the two questions were similar, generating a common set of themes and subthemes. Effective strategies and ideas for improving communication and decision-making about goals of care clustered under five themes: patient and family factors, communication between healthcare providers and patients, interprofessional collaboration, education, and resources. Subthemes highlighted core elements of shared decision-making.

**Conclusions:**

Translating our findings into multifaceted interventions that consider patient and family factors, address knowledge gaps, optimize resource utilization, and facilitate communication and collaboration between patients, families and healthcare providers may improve communication and decision-making about goals of care.

**Electronic supplementary material:**

The online version of this article (doi:10.1186/s12904-015-0035-x) contains supplementary material, which is available to authorized users.

## Background

With an aging population [1], planning for care at the end of life (EOL) is increasingly important. The greater availability of life-sustaining technologies presents health care providers, patients and caregivers with important and complicated decisions to make during the stress of acute illness [2].

Healthcare providers often do not discuss goals of care with seriously ill hospitalized patients [3, 4] or they approach these discussions inadequately [5, 6], contributing to provision of high intensity life support in the final months of life, even when patients and caregivers prefer treatments focused upon comfort and quality of life [7–11].

Addressing the gap between the care provided and that desired, and providing high quality patient-centred EOL care will require improved communication and decision-making about goals of care. For seriously ill hospitalized patients, goals of care conversations include deliberation and decision-making about the use or non-use of life-sustaining treatments [12]. While many previous studies of EOL communication have focused on barriers [13, 14], a solutions-oriented focus can also generate important insights [15, 16]. Recognizing that effective initiatives to overcome perceived barriers can be found within a community itself, the objective of the current multi-centre, cross-sectional survey was to elicit hospital-based healthcare providers’ strategies for effective goals of care discussions and decision-making. This study of healthcare provider experiences follows our previous exploration of patient and family perspectives on barriers and facilitators to advance care planning (the Audit of Communication, CarE Planning, and DocumenTation (ACCEPT) study [17].

## Methods

### Design and setting

This cross-sectional study, conducted from September 2012 to March 2013, involved a self-administered questionnaire about effective goals of care communication and decision-making in relation to medical interventions desired in serious illness; it was distributed to hospital-based clinicians in general internal medical teaching units (MTUs) at 13 academic centres in six Canadian provinces [18]. Research ethics approval was obtained at each participating site (Additional file 1).

### Participants

All eligible attending physicians, residents, and nurses were invited to participate, with the exception of centres with more than 50 nurses, where computer generated random number lists selected a sample of 50 nurses. Eligible participants were: (i) MTU attending physicians; (ii) postgraduate internal medicine residents (visiting residents were excluded); (iii) nurses (registered nurses, licensed practical nurses, registered practical nurses) employed full-time or part-time in a MTU at a participating centre. While many healthcare providers communicate with patients in hospital, we chose to focus upon nurses, residents and physicians for reasons of feasibility and because these providers are involved in the clinical care of every patient in a MTU while other providers are less frequently involved, depending on a patient’s needs.

### Study procedures

Details of questionnaire development and distribution have previously been described [18]. Briefly, a questionnaire based on previous literature was developed and refined in consultation with a multidisciplinary group of experts, followed by pilot-testing to produce a survey instrument with face and content validity, and clinical sensibility (Additional file 2) [18]. English and French versions of the questionnaire were developed in paper-based and online formats. Questionnaires were distributed to all eligible healthcare providers, with up to two reminders for non-responders. Consent was implied by return of a completed survey. The following definition was given to survey participants: “We define communication and decision‐making about goals of care as a conversation in which, ideally, a patient or family member and the healthcare team establish the goals of treatment (e.g., cure, prolongation of life, comfort) and agree upon the types of life sustaining technology that will (or will not) be used to achieve those goals (e.g., CPR, mechanical ventilation, dialysis, intensive care unit admission, feeding tubes, or intravenous hydration).”

In this paper, we report on free-text responses to two open-ended questions from the larger survey (provided as online supplemental material):

1. “What specific suggestions do you have about ways to overcome these barriers [rated as very or extremely important in the preceding section] and make it easier for health care providers to talk with patients and their family members about goals of care?”
2. “What is currently working well to promote communication and decision-making about goals of care between health care providers and patients and their family members?”

### Analysis

We used descriptive statistics to summarize respondent demographics, computing the mean and standard deviation for continuous variables and proportions for categorical variables. To analyze free text responses, bilingual investigators first translated French responses into English. Some respondents only described barriers rather than providing strategies for improvement or strategies that were currently effective; these comments were excluded from analysis as they did not pertain to the research question for this paper. Thematic content analysis was used to analyze free text responses [19]. Two of the study investigators (NS, AR) independently reviewed the free text responses and inductively developed a preliminary coding framework through multiple readings. These initial findings were discussed with a third investigator (JY) to reach consensus on key themes and subthemes. As the codes for responses to the two questions (“suggestions for overcoming barriers” and “currently effective strategies”) were identical, we summarized the findings with a common set of key themes and subthemes.

## Results

### Participants

**Fig. 1:**
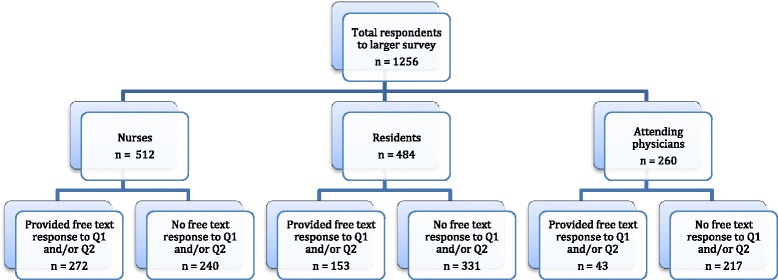
Flow diagram of respondents to free text survey questions*. *Question 1 (Q1) – “What specific suggestions do you have about ways to overcome these barriers [rated as very or extremely important in the preceding section] and make it easier for health care providers to talk with patients and their family members about goals of care?” Question 2 (Q2) – “What is currently working well to promote communication and decision-making about goals of care between health care providers and patients and their family members?”

Questionnaires were returned by 1,256 of 1,617 eligible healthcare providers, with an overall response rate of 78 % for the larger survey that included both the open and closed-ended responses (512 of 646 nurses [79 %], 484 of 634 residents [76 %], 260 of 337 physicians [77 %]). A free-text response to Question 1 and/or Question 2 (the open-ended survey questions) was provided by 468 (37 %) of the 1,256 healthcare providers who responded to the larger survey (272 of 512 nurses [53 %], 153 of 484 residents [32 %], 43 of 260 physicians [17 %], Fig. 1).

Demographic information of free text question respondents is presented in Table 1. The mean age of respondents was 36.3 years, 27.7 years, and 46.3 years for nurses, residents, and attending physicians, respectively. Of the nurse, resident, and attending physician respondents, 91.5 %, 39.9 %, and 39.5 % were female, respectively. Over 85 % of respondents had completed their basic clinical education in Canada. The majority of all respondents (78.2 %) had not received prior formal teaching in goals of care discussions.

**Table 1:** Demographic information for study participants who provided a free text response to Question 1 and/or Question 2*

Respondents		Nurses	Residents	Attending physicians
		(*n* = 272)	(*n* = 153)	(*n* = 43)
Age (years)	Mean ± SD (n)	36.3 ± 10.5 (255)	27.7 ± 3.0 (151)	46.3 ± 11.1 (42)
Sex	Male	21 (7.7 %)	92 (60.1 %)	25 (58.1 %)
	Female	249 (91.5 %)	61 (39.9 %)	17 (39.5 %)
	Missing	2 (0.7 %)	0	1 (2.3 %)
Years worked in practice	Mean ± SD (n)	8.8 ± 8.9 (263)	n/a	14.2 ± 10.3 (41)
Basic clinical (MD or nursing) education completed in Canada	(n)	238 (87.5 %)	138 (90.2 %)	37 (86.0 %)
Previous communication skills training	No	234 (86.0 %)	98 (64.1 %)	34 (79.1 %)
	Yes	33 (12.1 %)	53 (34.6 %)	9 (20.9 %)
	Missing	5 (1.8 %)	2 (1.3 %)	0 (0.0 %)

*Not all participants provided all demographic information

### Overview of themes

Five themes emerged from the free-text response analysis: patient and family factors, communication between healthcare providers and patients, interprofessional collaboration, education, and resources. Each main theme contained several subthemes. Some subthemes were common across all three healthcare provider groups (nurses, residents, and attending physicians), whereas others were specific to only one or two groups. The themes and subthemes are described below and summarized in Table 2, along with examples and illustrative quotes.

**Table 2:** Currently effective practices and ideas to improve communication and decision-making about goals of care*

Theme	Subthemes	Illustrative quotes
Patient and family involvement factors	Advanced illness as a trigger for EOL discussions	Evidence of advanced illness (Resident)
	Patient decisional capacity, substitute decision-making, family spokesperson	Knowing who substitute decision makers are when applicable (Nurse)
		Clearly identify who is the substitute decision maker on the first day (Resident)
	Care provided at EOL – family involvement in care provision, remaining in community location	Re-establishing the importance of care provided in nursing homes therefore no admit/return [to hospital] (Nurse)
		Involve the family in patient care… they’re part of the team (Nurse)
Patient-family-healthcare provider (HCP) communication	Timing of communication – pre-hospital, early initiation, prior to deterioration, in stages, allowing time, expectations, reassessment, checkpoint prior to discharge	End of life care discussions need to take place sooner… too often these discussions take place at onset or during an episode or code blue (Nurse)
		… in a non-rushed manner so goals/decisions can be discussed and explored adequately (Resident)
		Build a GOC discussion into the daily work flow of the medical teaching unit, for example, expected by day three (Physician)
	Continuity of communication between patients, families and healthcare providers, continuity of care provided by physicians (community, hospital), nursing, social workers, occupational therapists, physiotherapists	Having one physician designated for communication with a family member representative (Nurse)
		Patient’s general practitioner and primary care Respirologist to come see as in-patient (Resident)
	Process of communication – meetings, all stakeholders, initiator, willingness, set time, enough time, clear language, agenda, normalized, rapport, empathy, honesty, respectful, realistic, tailored, frequent updates, reassessment, consistent message, check understanding, agreement	Family meetings I find really help discussions and plans… multidisciplinary opinions, gives the family a bigger picture (Nurse)
	Content of communication – medical facts, diagnosis, prognosis, options, perspectives, experiences, quality of life, functional capacity, beliefs, goals, values, preferences, expectations, emotions, recommendation, contact information	An open and honest approach with two-way dialogue (Physician)
		Developing a rapport/relationship with family in order to gauge how much they understand about prognosis and then understanding what they think the medical care can provide (Resident)
		Improved functional history on admission to assist with prognostic assessment & realistic risk/benefit assessment of interventions (Resident)
Interprofessional collaboration	Approach to interprofessional communication – respect, support, dialogue, meetings, bullet rounds, pre-briefing in advance of family meetings, whiteboard, common goals, primary care team involvement	Rounds among the staff have also been helpful in communicating the plan of care among physicians, nurses, and allied staff (Nurse)
		Multidisciplinary meetings to establish a common understanding of prognosis and direction of patient care (Resident)
		The teamcare model helps to ensure all team members are on the same page and can support each other through these conversations (Nurse)
	Role clarity – clarify healthcare provider responsible for GOC discussions, expand nursing role, coordinate health care service delivery	Clear information as to whose responsibility it is to discuss GOC (Nurse)
		Expanding the scope of nurses in hospital to discuss life sustaining therapies (Nurse)
	Documenting GOC, substitute decision maker, family, name and designation of MD who discussed – clear documentation, standardized forms, in standardized location, include GOC designation on daily patient list	Clearly written [GOC] documentation (standardized) to avoid misinterpretation. (Nurse)
		Clear documentation in the chart about the plan, what the patient has been informed of with respect to their plan of care and current status (Nurse)
Education	Societal/public awareness of advance care planning, GOC, life-sustaining therapy, financial cost of healthcare – national dialogue, education, patient stories	Information sessions for the general public to help change the discourse surrounding death and dying (Physician)
		A national dialogue needs to be started with public at large to get people talking about advance directives and sharing that info with loved ones so families are not left feeling they have all the guilt & responsibility… (Nurse)
	Patient-family education about initiating GOC discussions	Distribute a booklet of information to patients/families to educate them prior to a GOC discussion (Physician)
	GOC, life-sustaining therapy, financial cost, ICU outcomes – pamphlets, videos, online resources	
		I think that education for patients and families regarding the realities of life sustaining therapies is required (Resident)
	Healthcare provider education about GOC, palliative care, EOL care, conflict, culture, prognostication – physician leadership, interprofessional learning, resident teaching and role modeling through undergraduate and postgraduate medical education and continuing medical education courses	Resident education and PGY-1 [teaching sessions] on communication have been very helpful (Resident)
		Training for physicians or other healthcare providers to help counsel patients and families better (Physician)
Resources	Healthcare personnel – increase healthcare provider to patient ratios, physician availability, increase attending physician involvement, unit champions, nurse liaisons	Physician availability to speak with family members (Nurse)
		Have unit champions who are versed in end of life care and can act as a resource for their team (Nurse)
	Consultant involvement – Palliative care, Oncology, Geriatrics, Psychology, Social Work, Spiritual Care, Occupational Therapy, Physiotherapy, Nurse Practitioners, Ethics, cultural expert	Ensure appropriate support staff consulted as early as possible (e.g.: palliative care, social work) (Nurse)
		The social workers can be quite helpful in facilitating family meetings to talk about goals of care (Resident)
	Physical space – quiet locations with privacy	Having 'family rooms' on units where discussions can be held (Resident)
		Need to have private rooms to discuss goals of care with patients and families (Resident)
	Access to documents – advance directives, specialist clinic notes, patient to carry documentation	Lack of information about prognosis would be improved by having proper access to specialists’ notes (Resident)
		Written advance directive travel with patient to hospital (Resident)
	Translators – availability, training in GOC discussions, available 24 h, mobile phones	Utilizing translation services (rather than family members) (Nurse)
		Interpreters who are well versed and familiar with these conversations need to be available at all times (Resident)
	Organizational support – patient-centred philosophy, organizational policies for GOC discussions, policy to address GOC discussions at admission, institutional culture of GOC discussions, advance care planning/GOC guideline development, remuneration, help with conflict resolution	A patient-centred facility give[s] patients and their families encouragement to be more active in their own care, which helps health care providers speak more regarding goals (Nurse)
		It is part of the culture to address this with nearly every admitted patient (Resident)

*GOC* goals of care; *QOL* quality of life; *PGY-1* post-graduate year 1 (first year resident)

*Table 2 presents a synthesis of both currently effective practices and potential ideas for improving goals of care discussions, as described in free-text responses to the survey

#### Patient and family involvement

Subthemes directly related to the patient and family included advanced illness, substitute decision-making, and philosophies about location of EOL care. Advanced illness was perceived as a trigger for healthcare providers to initiate goals of care discussions and help patients identify a substitute decision-maker. Nurses advocated for supporting family involvement in providing EOL care in the hospital and at home.

#### Communication between healthcare providers, patients and family

Healthcare providers identified attention to the timing, content, process, and continuity of communication between healthcare providers, patients, and families as an important mechanism to improve the quality of goals of care discussions and decision-making.

With respect to timing, all groups emphasized that early communication between healthcare providers, patients, and families was important. Early communication could facilitate building rapport, determine the family spokesperson and substitute decision-maker, identify advance directives, and help patients and family better understand diagnoses, treatment options, and prognosis in the first days of hospitalization. Many free-text question respondents felt that initiating goals of care discussions within the first twenty-four hours of hospital admission is important; some healthcare providers in each group mentioned that patients and families need time to make these complex decisions, while others emphasized that healthcare providers need to readdress goals of care as patient condition improves or worsens.

Continuity of care within and across healthcare settings was identified as enabling difficult discussions. Specific strategies cited included involving community care physicians with longitudinal doctor-patient relationships in goals of care discussions, and consistent patient assignments in the hospital.

In describing currently effective approaches and ideas for improving goals of care discussions, respondents noted several issues related to the content and process of communication. Content issues included eliciting and exploring patient and family experiences, values and preferences; providing guidance to patients and families around goals of care decision-making; and making a recommendation tailored to the patient’s medical condition, prognosis and values. Process considerations included building rapport; ensuring sufficient time for discussion; use of clear language; assessment of patient and family understanding; achieving a shared understanding; and respecting patient values and preferences. Being honest and realistic in discussing goals of care and prognosis was also emphasized.

#### Interprofessional collaboration within and across care settings

All groups, and nursing staff in particular, highlighted the effectiveness of a collaborative, interprofessional approach to discussing and determining goals of care. Responses clustered into three subthemes: consistency of interprofessional communication, role clarity, and documentation.

Approaches to interprofessional collaboration included multidisciplinary family meetings and healthcare team meetings; nurses and residents specified the importance of attending physician involvement in these meetings. Nurses commented that dialogue between team members facilitated establishing plans of care, consistent messaging to patients and family, and coordinating services to achieve patient goals of care. Team support through reminders and communication between hospital and community healthcare providers was also described.

The need for clarifying the role of both individual and groups of healthcare providers in discussing goals of care was also stressed. For example, should addressing goals of care be the role of nurses, residents, or attending physicians? Some nurses desired greater involvement in the process, and proposed expanding nursing roles in discussing and determining goals of care.

All groups commented that documenting goals of care discussions and decisions would facilitate future healthcare provider awareness of the content and rationale for decisions made. A standardized form for documentation placed in a consistent section of the patient chart would enable consistency.

#### Education

Education of the public, patients and families, and healthcare providers about advance care planning and goals of care were core subthemes within the education theme. Suggested societal and patient/family education strategies included dialogue, informational brochures, and video clips of life support interventions accompanied by patient and family narratives of intensive care and chronic critical illness experiences.

Helpful strategies to establish expectations that goals of care be addressed included hospital policies and increasing physician awareness of the importance of goals of care discussions. Residents mentioned undergraduate and postgraduate medical education initiatives improved their comfort with these discussions, along with experiential learning during clinical rotations. Respondents specified that courses and informal learning through role-modeling and feedback could improve knowledge and skills related to communication overall, cultural diversity, advance care planning, goals of care discussions, and palliative care. Residents specifically valued attending physicians' involvement in patient care in general, and goals of care discussions in particular.

#### Resources

Each healthcare provider group identified the importance of directing resources towards facilitating goals of care discussions. Subthemes included document access, personnel, physical space, and organizational support. Residents in particular hoped for easier access to community practitioner documentation of advance care planning discussions and prognostication. Nursing staff and residents mentioned attending physician availability and sufficient time as conditions for effective goals of care discussions. All groups mentioned that higher healthcare provider to patient ratios would allow more time for family meetings and complex discussions. A facilitating role for consultants with expertise in EOL communication, and increased availability of interpreters were also described. Finally, nurses perceived a patient-centred focus at an organizational level as important, with policies and procedures that foster improved goals of care communication and decision-making.

## Discussion

Through qualitative analysis of free text responses to open-ended questions in a national, multicentre survey, we have described hospital-based healthcare providers’ perspectives on strategies to improve goals of care discussions and decision-making. These strategies clustered into five overall themes: patient and family involvement, healthcare provider communication with patients and families, interprofessional communication, education, and resources.

Our study focused on the positive, seeking to identify currently effective processes that can be further developed and expanded upon and ideas for improving current approaches, rather than focusing on negative experiences and challenges. Concepts mentioned as potential solutions by some participants were described by others as currently effective processes. Considering the multidisciplinary and multicentre nature of our study, this overlap may reflect different perspectives between individuals, or different practices between hospital units or institutions. A better understanding of this overlap could facilitate targeted interventions. For example, if one centre’s ideas for improvement are currently implemented elsewhere, sharing resources and experiences may both facilitate and expedite knowledge translation.

The concept of shared decision-making can be identified within all themes described in our study. Previous research has reported that patients and families value shared decision-making in establishing goals of care [6], while gaps exist between the care patients desire and the goals documented in the medical record [5, 17]. Healthcare providers in our study also described many concepts related to shared decision-making in elaborating the ideal content and process of goals of care communication (Table 2), yet challenges in translating this knowledge into decision-making in clinical practice remain [18]. Decision-making about goals of care in the acute care setting introduces numerous challenges for shared decision-making [20]. Time for making decisions may be limited, long term relationships have not been established, values are diverse, emotions are strong, and discussions and decisions have a profound impact on care provided and subsequent outcomes. With contemporary interprofessional models of healthcare delivery, communication amongst healthcare providers and between the patient/family, and different healthcare providers at various time points adds to the complexity of shared decision-making [20].

This study represents a subset of data from a larger survey [18]. The quantitative aspects of the larger survey focused on healthcare provider perceptions of barriers to discussing goals of care. Patient and family-related barriers were ranked as most important, followed by barriers related to communication between patients, family and healthcare providers; interprofessional collaboration, education and resources were ranked as less important. The current paper provides complementary data, where more ideas for improvement were focused on those factors that had been ranked as less important in the quantitative aspects of the survey. These findings may reflect survey design, in that more of the barrier-seeking questions in the larger survey were related to patient and family factors [18], or healthcare providers may have ranked factors beyond their locus of control as less important. An alternate explanation is the tendency toward positive self-regard, perceiving oneself and associated social groups more positively [21]. Positive self-regard may interfere with identifying barriers, whereas asking about effective strategies and ideas for improvement elicit favorable frames and remove personal attributions, respectively. This would have implications for the language used in future quantitative and qualitative research – framing questions in both problem-focused and solution-focused terms may provide more representative appraisals.

### Integrating the perspectives of healthcare providers, patients and families

**Fig. 2:**
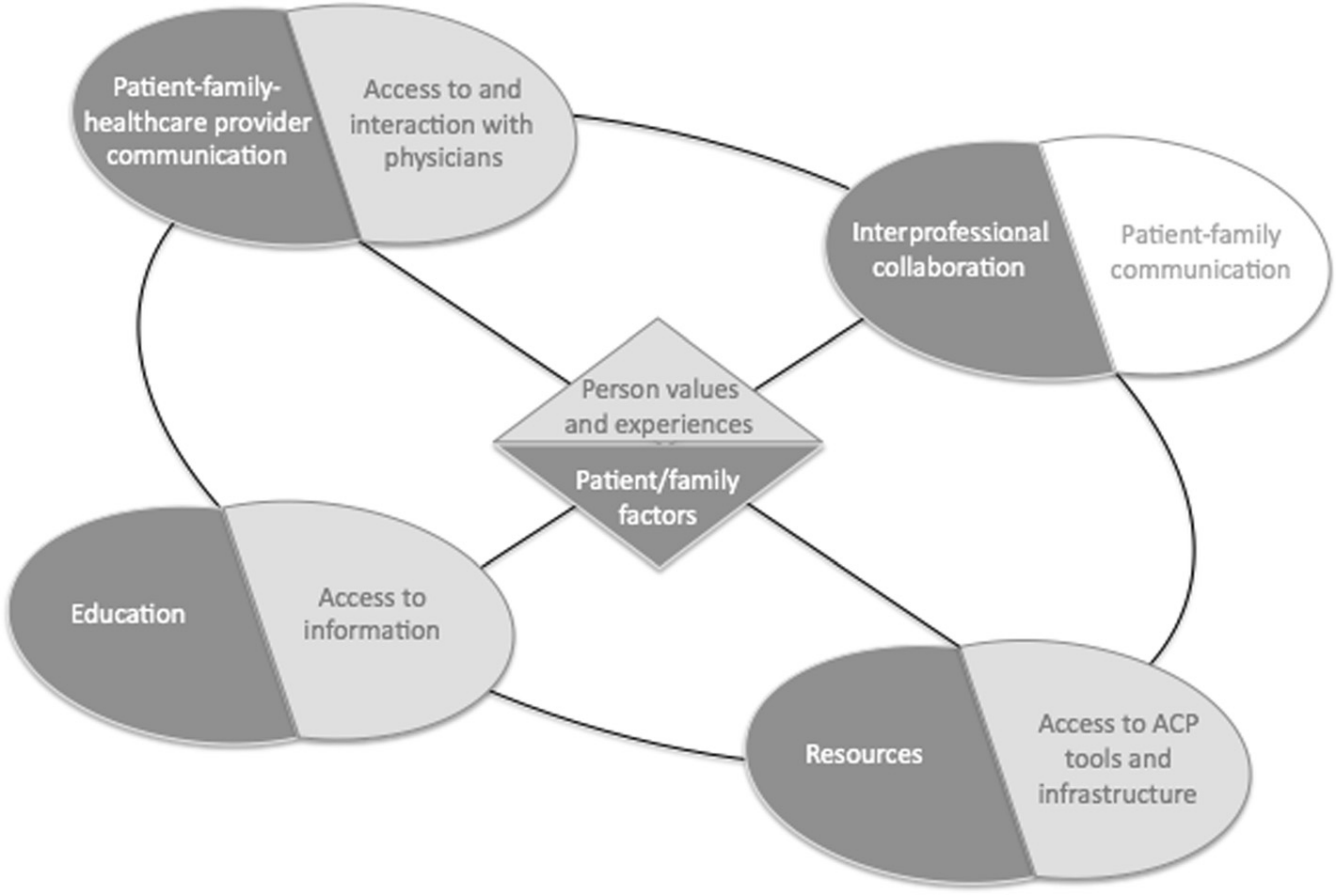
Factors facilitating goals of care discussions and decisions from the perspectives of healthcare providers (dark grey, as identified in the current study) and patients and families (light grey, as reported in ACCEPT). All of the themes in DECIDE correspond to one of the categories in the ACCEPT study, with the exception of interprofessional communication; an analogous theme from a patient perspective could be represented as patient-family communication (white)

While the current study focused on healthcare provider experiences, the recent multicentre ACCEPT study explored patient and family perspectives on barriers and facilitators of advance care planning [22]. Notably, many of the themes and subthemes in the two studies are aligned, as illustrated in Fig. 2. While there was not a category corresponding to our “Interprofessional collaboration” theme in the ACCEPT data, we propose that communication between patients and families could be represented as an analogous theme.

This alignment between patient and family needs and healthcare provider strategies for improving goals of care discussions is encouraging and may suggest that healthcare providers are sensitive to patient and family needs. However, if this was true, why do needs remain unmet? Perhaps the barriers outweigh the current potential for change. Change within the healthcare system is a complex process, influenced by individual and organizational readiness, perceived capacity to change, and contextual receptivity [23]. In proceeding with interventions to support change, we must be mindful that the thematic concepts we identified represent generalizations at a point in time; divergent perspectives between patients, families and healthcare providers within the systems through which they interact certainly exist. Identifying and responding to divergent needs by flexibly and dynamically adapting interventions to contextual nuances will be essential for program success.

### Practical implications

To impact patient care, insights about effective strategies for goals of care discussions will need to be translated into clinical practice. Our study describes the initial steps of knowledge creation – knowledge inquiry, identification of facilitators, and knowledge synthesis [24]. Creatively adapting this knowledge to local contexts will be the next step in knowledge translation, requiring identification, implementation, adaptation, and evaluation [24] of tools and programs to improve shared decision making about goals of care within the interprofessional setting of acute care medicine. A multidimensional approach that includes societal, healthcare provider and interprofessional education about advance care planning and goals of care will be important. Evidence-based guidance for communication with families in the ICU setting exists [25]. The literature also describes promising tools to support shared decision making about goals of care, including the internet-based PREPARE and other advance care planning decision aids [26, 27]; goals of care video decision aids [28]; Team Strategies and Tools to Enhance Performance and Patient Safety (TeamSTEPPS) for interprofessional collaboration and teamwork [29]; the Speak Up campaign (Canadian Hospice Palliative Care Association) [30] and The Conversation Project for public education (Institute for Healthcare Improvement) [31]; and Conversations Matter (Alberta Health Services) [32] for both public and healthcare provider education.

### Strengths and limitations

Strengths of our study include the multicentre data set involving multiple healthcare providers, with diversity in geography, language (French and English), and culture allowing generalizability across the Canadian context. Triangulation of data from nurses, residents and attending physicians adds additional rigour to our findings. Finally, open-ended questions elicited participant expression of ideas, affording a more elaborate description than can be obtained from closed-ended survey options, where investigator synthesis of pre-existing literature constrains findings.

One of the limitations is in the nature of free-text data, where ideas cannot be explored, clarified, or expanded upon, as would be possible with interviews and focus groups. Another limitation is our focus on physicians and nurses in the hospital setting; community-based practitioners, social workers, counselors and spiritual care providers would likely identify different approaches and ideas. Their views, as well as a greater number of free-text responses from attending physicians in the study, may have allowed for a broader range of perspectives on the issues. Response to free-text questions was optional; we cannot discern from our data why physicians were less inclined to respond to these questions than nurses or residents. Potential reasons include fatigue at the end of a long questionnaire, lack of ideas, or the perception that their opinions were sufficiently expressed in the closed-ended section of the questionnaire. Finally, while we did not elicit patient or family perspectives, complementary patient and family data exist in the literature [17, 20].

## Conclusion

Overall, our findings suggest that enhancing patient and family involvement, communication between patients, families and healthcare providers, interprofessional collaboration, educational initiatives, and resource availability may improve discussions and decision-making about goals of care for medical interventions among seriously ill patients in hospital. Ongoing developments in these areas may be facilitated by a range of promising interventions, such as internet-based or video decision aids for advance care planning and goals of care determination, family meetings, teamwork training, societal and healthcare provider educational interventions, and quality improvement initiatives. Further consideration, tailored implementation, and evaluation of such interventions are warranted.

## Additional files

Additional file 1:
**Ethics committees at participating research centres.** (PDF 35 kb) Additional file 2:
**Decision-Making About Goals of Care for Hospitalized Medical Patients Questionnaire – Physician version.** (PDF 145 kb)

## Abbreviations

ACCEPT: Audit of Communication, CarE Planning, and DocumenTation
EOL: End of life
MTU: Medical teaching unit
GOC: Goals of care
QOL: Quality of life
PGY: Postgraduate year
