# Supplementary material for: Strategies for effective goals of care discussions and decision-making: perspectives from a multi-centre survey of Canadian hospital-based healthcare providers
Source: BMC Palliat Care. 2015 Aug 19;14:38. doi: 10.1186/s12904-015-0035-x (PMC4544790; doi:10.1186/s12904-015-0035-x)
Supplement: Additional file 1: — Ethics committees at participating research centres. (PDF 35 kb) [file 12904_2015_35_MOESM1_ESM.pdf]

Additional file 1 – Ethics committees at participating research centres

1. Kingston General Hospital, Health Sciences Research Ethics Board
2. Hamilton General Hospital, Hamilton Integrated Research Ethics Board
3. Juravinski Hospital, Hamilton Integrated Research Ethics Board
4. St. Joseph’s Hospital, Hamilton Integrated Research Ethics Board
5. Toronto General Hospital, University Health Network Research Ethics Board
6. Sunnybrook Health Sciences Centre, Research Ethics Board
7. Hôpital du Sacré-Coeur de Montréal, Comité d’Éthique de la Recherche
8. Université de Sherbrooke, Comité d’Éthique de la Recherche
9. McGill University Health Centre, Research Ethics Board
10. University of British Columbia, Clinical Research Ethics Board
11. University of Calgary, Conjoint Health Research Ethics Board
12. University of Manitoba, Research Ethics Board
13. Memorial University of Newfoundland, Interdisciplinary Committee on Ethics in Human Research.
