# Supplementary material for: Strategies for effective goals of care discussions and decision-making: perspectives from a multi-centre survey of Canadian hospital-based healthcare providers
Source: BMC Palliat Care. 2015 Aug 19;14:38. doi: 10.1186/s12904-015-0035-x (PMC4544790; doi:10.1186/s12904-015-0035-x)
Supplement: Additional file 2: — Decision-Making About Goals of Care for Hospitalized Medical Patients Questionnaire – Physician version. (PDF 145 kb) [file 12904_2015_35_MOESM2_ESM.pdf]

MD - __ __ __

# Decision-Making about Goals of Care For Hospitalized Medical Patients

## PHYSICIAN VERSION

**We would greatly appreciate your participation in this questionnaire which addresses communication and decision-making about goals of care with seriously ill hospitalized patients and their families.**

**Goals of Care**

We define communication and decision-making about ***goals of care*** as a conversation in which, ideally, a patient or family member and the healthcare team establish the goals of treatment (e.g., cure, prolongation of life, comfort) and agree upon the types of life sustaining technology that will (or will not) be used to achieve those goals (e.g., CPR, mechanical ventilation, dialysis, intensive care unit admission, feeding tubes, or intravenous hydration).

In this questionnaire, we are interested in your perspective about:

(1) The importance of various barriers to communication and decision-making about goals of care with seriously ill hospitalized patients and their families
(2) Your suggestions to improve communication and decision-making about goals of care with these patients and their families
(3) Your perceived role and the role that others may play in communication and decision-making about goals of care with these patients and their families

- **This questionnaire will take approximately 15 minutes to complete.**
- **Please select the single best response to each question unless otherwise requested.**
- **All responses will remain confidential.**

# Section 1
# Barriers to Discussions about Goals of Care

**Scenario:** A 70 year old patient who has severe COPD (on home oxygen), is housebound, and requires assistance for most activities of daily living, is admitted to the medical ward under your care with an exacerbation of their COPD. The patient's acute symptoms have resolved. You are uncertain about the patient's goals of care and preferences regarding the use (or non-use) of life sustaining technology.

## Barriers Related to the Patient/Family

1. Reflecting on your most recent month on an acute medical ward, for patients such as the one described in the above scenario, please rate the importance of the following barriers in preventing **you** from talking to them and/or their family members about the patient's goals of care. As you rate the importance of each barrier, consider both the magnitude of the barrier and its frequency and circle the best response from 1 = Extremely Unimportant Barrier to 7 = Extremely Important Barrier.

| Extremely Unimportant Barrier | Very Unimportant Barrier | Somewhat Unimportant Barrier | Neither Important nor Unimportant Barrier | Somewhat Important Barrier | Very Important Barrier | Extremely Important Barrier |
|---|---|---|---|---|---|---|
| a) Patient does not have an advance directive | | | | | | |
| 1 | 2 | 3 | 4 | 5 | 6 | 7 |
| b) Patient has an advance directive but it lacks sufficient detail | | | | | | |
| 1 | 2 | 3 | 4 | 5 | 6 | 7 |
| c) Patients' difficulty accepting their poor prognosis | | | | | | |
| 1 | 2 | 3 | 4 | 5 | 6 | 7 |
| d) Patients' difficulty understanding the limitations and complications of life sustaining therapies | | | | | | |
| 1 | 2 | 3 | 4 | 5 | 6 | 7 |
| e) Lack of patient capacity to make decisions about goals of care | | | | | | |
| 1 | 2 | 3 | 4 | 5 | 6 | 7 |
| f) Family members' difficulty accepting their loved one's poor prognosis | | | | | | |
| 1 | 2 | 3 | 4 | 5 | 6 | 7 |
| g) Family members' difficulty understanding the limitations and complications of life sustaining therapies | | | | | | |
| 1 | 2 | 3 | 4 | 5 | 6 | 7 |
| h) Lack of agreement amongst family members about goals of care | | | | | | |
| 1 | 2 | 3 | 4 | 5 | 6 | 7 |
| i) Language barriers | | | | | | |
| 1 | 2 | 3 | 4 | 5 | 6 | 7 |
| j) Differences in approach to discussing goals of care in some cultures | | | | | | |
| 1 | 2 | 3 | 4 | 5 | 6 | 7 |

## Barriers Related to the Role of Physicians

2. Reflecting on your most recent month on an acute medical ward, for patients such as the one described in the above scenario, please rate the importance of the following barriers in preventing **you** from talking to them and/or their family members about the patient's goals of care. As you rate the importance of each barrier, consider both the magnitude of the barrier and its frequency.

| | Extremely Unimportant Barrier | Very Unimportant Barrier | Somewhat Unimportant Barrier | Neither Important nor Unimportant Barrier | Somewhat Important Barrier | Very Important Barrier | Extremely Important Barrier |
|---|---|---|---|---|---|---|---|
| a) Uncertainty in estimating prognosis | **1** | **2** | **3** | **4** | **5** | **6** | **7** |
| b) Lack of training to have these conversations (e.g. not sure what to say, etc.) | **1** | **2** | **3** | **4** | **5** | **6** | **7** |
| c) Desire to avoid being sued | **1** | **2** | **3** | **4** | **5** | **6** | **7** |
| d) Desire to maintain hope | **1** | **2** | **3** | **4** | **5** | **6** | **7** |

## Barriers Related to the System or External factors

3. Reflecting on your most recent month on an acute medical ward, for patients such as the one described in the above scenario, please rate the importance of the following barriers in preventing **you** from talking to them and/or their family members about the patient's goals of care. As you rate the importance of each barrier, consider both the magnitude of the barrier and its frequency.

| | Extremely Unimportant Barrier | Very Unimportant Barrier | Somewhat Unimportant Barrier | Neither Important nor Unimportant Barrier | Somewhat Important Barrier | Very Important Barrier | Extremely Important Barrier |
|---|---|---|---|---|---|---|---|
| a) Lack of time to have conversations with patients/family | **1** | **2** | **3** | **4** | **5** | **6** | **7** |
| b) Lack of availability of substitute decision maker(s) | **1** | **2** | **3** | **4** | **5** | **6** | **7** |
| c) Uncertainty about who is the substitute decision maker | **1** | **2** | **3** | **4** | **5** | **6** | **7** |
| d) Lack of an appropriate location to maintain patient confidentiality and privacy | **1** | **2** | **3** | **4** | **5** | **6** | **7** |
| e) Insufficient remuneration for this activity | **1** | **2** | **3** | **4** | **5** | **6** | **7** |
| f) Lack of pre-existing relationship with patient/family | **1** | **2** | **3** | **4** | **5** | **6** | **7** |
| g) Lack of awareness of what other members of the care team have said | **1** | **2** | **3** | **4** | **5** | **6** | **7** |
| h) Disagreement among healthcare team about goals of care | **1** | **2** | **3** | **4** | **5** | **6** | **7** |

## Other Barriers

4. Reflecting on your most recent month on an acute medical ward, for patients such as the one described in the above scenario, please describe any **other barriers** in preventing **you** from talking to them and/or their family members about the patient's goals of care and rate their importance. As you rate the importance of each barrier, consider both the magnitude of the barrier and its frequency.

| **Extremely Unimportant Barrier** | **Very Unimportant Barrier** | **Somewhat Unimportant Barrier** | **Neither Important nor Unimportant Barrier** | **Somewhat Important Barrier** | **Very Important Barrier** | **Extremely Important Barrier** |
|---|---|---|---|---|---|---|

a) Other Barrier (please specify):________________________________________________________________

| | | | | | | |
|---|---|---|---|---|---|---|
| **1** | **2** | **3** | **4** | **5** | **6** | **7** |

b) Other Barrier (please specify):________________________________________________________________

| | | | | | | |
|---|---|---|---|---|---|---|
| **1** | **2** | **3** | **4** | **5** | **6** | **7** |

c) Other Barrier (please specify):________________________________________________________________

| | | | | | | |
|---|---|---|---|---|---|---|
| **1** | **2** | **3** | **4** | **5** | **6** | **7** |

d) Other Barrier (please specify):________________________________________________________________

| | | | | | | |
|---|---|---|---|---|---|---|
| **1** | **2** | **3** | **4** | **5** | **6** | **7** |

# Section 2
# Suggestions to Improve Communication and Decision-Making about Goals of Care

1. Reflecting on the barriers which you rated as **Very Important** or **Extremely Important** in Section 1, what specific suggestions do you have about ways to overcome these barriers and make it easier for health care providers to talk with patients and their family members about goals of care?

2. What is currently working well to promote communication and decision-making about goals of care between health care providers and patients and their family members?

# Section 3
# Health Care Providers' Role in Communication and Decision-Making about Goals of Care

**Please consider the same scenario as in Section 1:** A 70 year old patient who has severe COPD (on home oxygen), is housebound, and requires assistance for most activities of daily living, is admitted to the medical ward under your care with an exacerbation of their COPD. The patient's acute symptoms have resolved. You are uncertain about the patient's goals of care and preferences regarding the use (or non-use) of life sustaining technology.

Consider that the process of communication and decision making can be broken down into the following steps: initiating the conversation, exchanging information, coaching, and deciding.

## Willingness to Participate in Communication and Decision-Making about Goals of Care

| **Extremely Unwilling** | **Very Unwilling** | **Somewhat Unwilling** | **Neither Willing nor Unwilling** | **Somewhat Willing** | **Very Willing** | **Extremely Willing** |
|---|---|---|---|---|---|---|

1. Rate your willingness to <u>initiate discussions</u> about goals of care *with* patients such as these, and their families.

| | | | | | | |
|---|---|---|---|---|---|---|
| **1** | **2** | **3** | **4** | **5** | **6** | **7** |

2. Rate your willingness to <u>exchange information</u> (e.g., disclose diagnosis, prognosis and elicit values) *with* patients such as these, and their families, who are trying to reach a decision about goals of care.

| | | | | | | |
|---|---|---|---|---|---|---|
| **1** | **2** | **3** | **4** | **5** | **6** | **7** |

3. Rate your willingness to be a <u>decision coach</u> (clarifying values, assisting with weighing options for care, etc.) *for* patients such as these, and their families, who are trying to reach a decision about goals of care.

| | | | | | | |
|---|---|---|---|---|---|---|
| **1** | **2** | **3** | **4** | **5** | **6** | **7** |

4. Rate your willingness to participate in <u>making a final decision about</u> goals of care *with* patients such as these, and their families.

| | | | | | | |
|---|---|---|---|---|---|---|
| **1** | **2** | **3** | **4** | **5** | **6** | **7** |

## Participation of Health Care Professionals in Communication and Decision-Making about Goals of Care

For each category of hospital-based health care professionals listed below, please rate how acceptable you would find it for that group of individuals to be involved with each of the following activities:

5. **Initiating discussions** about goals of care:

| | Extremely Unacceptable | Very Unacceptable | Somewhat Unacceptable | Neither Acceptable nor Unacceptable | Somewhat Acceptable | Very Acceptable | Extremely Acceptable |
|---|---|---|---|---|---|---|---|
| a) Staff physician | 1 | 2 | 3 | 4 | 5 | 6 | 7 |
| b) Resident | 1 | 2 | 3 | 4 | 5 | 6 | 7 |
| c) Bedside nurse | 1 | 2 | 3 | 4 | 5 | 6 | 7 |
| d) Advance practice nurse (i.e., clinical nurse specialist or nurse practitioner) | 1 | 2 | 3 | 4 | 5 | 6 | 7 |
| e) Social worker | 1 | 2 | 3 | 4 | 5 | 6 | 7 |
| f) Other (specify): _______________________________ | 1 | 2 | 3 | 4 | 5 | 6 | 7 |

6. **Exchanging information** (e.g., disclose diagnosis, prognosis and elicit values):

| | Extremely Unacceptable | Very Unacceptable | Somewhat Unacceptable | Neither Acceptable nor Unacceptable | Somewhat Acceptable | Very Acceptable | Extremely Acceptable |
|---|---|---|---|---|---|---|---|
| a) Staff physician | 1 | 2 | 3 | 4 | 5 | 6 | 7 |
| b) Resident | 1 | 2 | 3 | 4 | 5 | 6 | 7 |
| c) Bedside nurse | 1 | 2 | 3 | 4 | 5 | 6 | 7 |
| d) Advance practice nurse (i.e., clinical nurse specialist or nurse practitioner) | 1 | 2 | 3 | 4 | 5 | 6 | 7 |
| e) Social worker | 1 | 2 | 3 | 4 | 5 | 6 | 7 |
| f) Other (specify): _______________________________ | 1 | 2 | 3 | 4 | 5 | 6 | 7 |

7. **Decision coach** (clarifying values, assisting with weighing options for care, etc.):

| Extremely Unacceptable | Very Unacceptable | Somewhat Unacceptable | Neither Acceptable nor Unacceptable | Somewhat Acceptable | Very Acceptable | Extremely Acceptable |
|---|---|---|---|---|---|---|
| a) Staff physician | | | | | | |
| **1** | **2** | **3** | **4** | **5** | **6** | **7** |
| b) Resident | | | | | | |
| **1** | **2** | **3** | **4** | **5** | **6** | **7** |
| c) Bedside nurse | | | | | | |
| **1** | **2** | **3** | **4** | **5** | **6** | **7** |
| d) Advance practice nurse (i.e., clinical nurse specialist or nurse practitioner) | | | | | | |
| **1** | **2** | **3** | **4** | **5** | **6** | **7** |
| e) Social worker | | | | | | |
| **1** | **2** | **3** | **4** | **5** | **6** | **7** |
| f) Other (specify): _________________________________ | | | | | | |
| **1** | **2** | **3** | **4** | **5** | **6** | **7** |

8. **Making a final decision** about goals of care:

| Extremely Unacceptable | Very Unacceptable | Somewhat Unacceptable | Neither Acceptable nor Unacceptable | Somewhat Acceptable | Very Acceptable | Extremely Acceptable |
|---|---|---|---|---|---|---|
| a) Staff physician | | | | | | |
| **1** | **2** | **3** | **4** | **5** | **6** | **7** |
| b) Resident | | | | | | |
| **1** | **2** | **3** | **4** | **5** | **6** | **7** |
| c) Bedside nurse | | | | | | |
| **1** | **2** | **3** | **4** | **5** | **6** | **7** |
| d) Advance practice nurse (i.e., clinical nurse specialist or nurse practitioner) | | | | | | |
| **1** | **2** | **3** | **4** | **5** | **6** | **7** |
| e) Social worker | | | | | | |
| **1** | **2** | **3** | **4** | **5** | **6** | **7** |
| f) Other (specify): _________________________________ | | | | | | |
| **1** | **2** | **3** | **4** | **5** | **6** | **7** |

# Section 4
# Personal Demographics

**1. What is your current position?**

- ❑ PGY-1
- ❑ PGY-2
- ❑ PGY-3
- ❑ PGY-4 or above
- ❑ Staff physician (If yes, how many years have you been in independent practice? _________ years)

**2. a) For residents, how many weeks of a Medical Teaching Unit rotation have you completed so far during your residency?**

__________ weeks

**2. b) For staff physicians, on average, how many weeks per year do you work on a Medical Teaching Unit?**

__________ weeks

**3. Where did you graduate from medical school?**

- ❑ Canada
- ❑ United States
- ❑ United Kingdom / Ireland / Australia / New Zealand
- ❑ Europe
- ❑ Asia
- ❑ Middle East
- ❑ Central or South America
- ❑ Africa
- ❑ Other: ___________________________________

**4. Have you ever worked on a formal palliative care consultation or inpatient service (includes resident rotation)?**

- ❑ Yes
- ❑ No

**5. Please indicate if you have formal specialty training in a field other than general internal medicine? (check all that apply)**

- ❑ Critical Care
- ❑ Respirology
- ❑ Cardiology
- ❑ Endocrinology
- ❑ Gastroenterology
- ❑ Geriatrics
- ❑ Hematology
- ❑ Infectious Diseases
- ❑ Medical Oncology
- ❑ Nephrology
- ❑ Rheumatology
- ❑ Other (please specify): _____________________

**6. Please rank your current level of skill in having goals of care discussions and making goals of care decisions with patients and families:**

| Limited (skill not developed) | Fair | Average (skill comparable to colleagues at same level) | Very Good | Expert (skill comparable to a palliative care physician) |
|---|---|---|---|---|
| **1** | **2** | **3** | **4** | **5** |

**7. Please rank your priority (1 to 5) for learning this skill:**

| Low Priority (not of interest or already mastered) | | | | High Priority (#1 on personal learning agenda) |
|---|---|---|---|---|
| **1** | **2** | **3** | **4** | **5** |

**8. a) Have you received any formal training regarding communication with patients and families about goals of care?**

- ❑ Yes ➔ If yes, please answer question 8b
- ❑ No

**8. b) Please rate the quality of the formal training you received regarding communication with patients and families about goals of care**

| Extremely Low | Very Low | Moderately Low | Neither High nor Low | Moderately High | Very High | Extremely High |
|---|---|---|---|---|---|---|
| **1** | **2** | **3** | **4** | **5** | **6** | **7** |

**9. What is your age?**

_________ years

**10. What is your sex?**

- ❑ Male
- ❑ Female

**11. Do you see yourself as:**
(Check <u>only</u> one)

- ❑ White
- ❑ South Asian (e.g., East Indian, Pakistani, Sri Lankan, etc.)
- ❑ Chinese
- ❑ Black
- ❑ Filipino
- ❑ Latin American
- ❑ Arab
- ❑ Southeast Asian (e.g., Vietnamese, Cambodian, Malaysian, Laotian, etc.)
- ❑ West Asian (e.g., Iranian, Afghan, etc.)
- ❑ Korean
- ❑ Japanese
- ❑ Native American
- ❑ Other: ____________________________________

**12. Please indicate your religious background:**
(Check <u>only</u> one)

- ❑ Roman Catholic
- ❑ Protestant Christian
- ❑ Orthodox Christian
- ❑ Other Christian
- ❑ Muslim
- ❑ Jewish
- ❑ Buddhist
- ❑ Hindu
- ❑ Sikh
- ❑ No religious affiliation
- ❑ Other: ____________________________________

**13. How important is spirituality or religion in your life?**

| Extremely Unimportant | Very Unimportant | Somewhat Unimportant | Neither Important nor Unimportant | Somewhat Important | Very Important | Extremely Important |
|---|---|---|---|---|---|---|
| **1** | **2** | **3** | **4** | **5** | **6** | **7** |

**14. Have you had personal experience with a close family member or friend being hospitalized in an intensive care unit?**

- ❑ Yes
- ❑ No

## Thank you for your participation in this survey!

Please return this survey in the provided envelope.
